# Adapting screening, brief intervention, and referral to treatment to tobacco: a hospital trial of warm handoffs for smoking cessation

**DOI:** 10.1186/1940-0640-7-S1-A59

**Published:** 2012-10-09

**Authors:** Kimber Richter, Biatriz Carlini, Jamie Hunt, Babalola Faseru, Laura Mussulman

**Affiliations:** 1 Department of Preventive Medicine and Public Health, University of Kansas Medical Center, Kansas City, KS, USA; 2 Alcohol and Drug Abuse Institute, University of Washington, Seattle, WA, USA; 3 Preventive Medicine & Public Health Tobacco Research Group, University of Kansas Medical Center, Kansas City, KS, USA

## 

Post-discharge support is key to effective treatment for hospitalized smokers. The few hospitals that systematically address tobacco refer smokers via fax to tobacco quitlines, yet few smokers enroll. “Warm handoff” (direct referral by one provider to another provider) is used in some cases of tobacco screening and brief intervention (SBI) to link patients with treatment, but little data exists on process or outcomes. We present pilot outcomes and early implementation data on an ongoing randomized controlled trial (RCT). Recruitment began in July 1, 2011. The purpose of the trial (Enhancing Quitline Utilization by Inpatients [EQUIP]) is to determine the effectiveness and cost-effectiveness of warm handoff versus fax referral for linking hospitalized smokers with tobacco quitlines. The EQUIP trial is a two-arm RCT funded by the National Institutes of Health (trial registration: NCT01305928) in which smokers who wish to quit permanently after discharge are randomized to either fax referral (standard in-hospital intervention with fax-referral for counseling post-discharge) or warm handoff (brief in-hospital intervention and immediate staff call to the state quitline and transfer of the call to the patient’s mobile or bedside hospital phone for enrollment and an initial counseling session). Outcomes, including costs, are assessed at one, six, and 12 months following baseline. We hypothesize that warm handoff will outperform fax referral in terms of enrollment in services, cessation, and cost-effectiveness. This study explores what alcohol and other drug trials have done to strengthen handoffs and the evidence for efficacy; how EQUIP compares with AOD handoffs, and what pitfalls might be expected; and whether tobacco SBI and referral to treatment would or could be incorporated into alcohol and other drug SBI and referral to treatment.

